# Are very thin patients at a higher risk of complications when submitted to percutaneous nephrolithotomy?

**DOI:** 10.1590/S1677-5538.IBJU.2024.0341

**Published:** 2024-08-25

**Authors:** Priscila Kuriki Vieira Mota, Daniel Beltrame Ferreira, Rafael Felisberto Dias Florencio, David Jacques Cohen, Rodrigo Perrella, Carlos Alfredo Batagello, Claudio Bovolenta Murta, Joaquim Francisco de Almeida Claro, Fabio C. Vicentini

**Affiliations:** 1 Hospital Brigadeiro Divisão de Urologia São Paulo SP Brasil Divisão de Urologia, Hospital Brigadeiro, São Paulo, SP, Brasil;; 2 Hospital Militar de Área de São Paulo Divisão de Urologia São Paulo SP Brasil Divisão de Urologia, Hospital Militar de Área de São Paulo, São Paulo, SP, Brasil;; 3 Hospital das Clínicas Divisão de Urologia São Paulo SP Brasil Divisão de Urologia, Hospital das Clínicas, São Paulo, SP, Brasil

**Keywords:** Urolithiasis, Nephrolithotomy, Percutaneous, Body Mass Index

## Abstract

**Purpose:**

To assess the impact of thinness on the outcome of the percutaneous nephrolithotomy (PCNL).

**Materials and Methods:**

A matched case–control study was performed using a prospectively collected database of all patients who underwent PCNL between June 2011 and October 2021. The patients were stratified into two groups according to their phenotypic characteristics, arbitrarily defined according to their body mass index (BMI): <20 kg/m^2^ (Group 1, very thin patients, G<20) and ≥25 kg/m^2^ (Group 2, non-thin patients, G≥25). Patients were randomly matched based on Guy's Stone Score (GSS) according to case complexity at a ratio of 1:3.

**Results:**

A total of 204 patients were enrolled in this study: 51 patients (G<20) and 153 controls (G≥25). Complications occurred in 15.2% of the patients, with 5.4% of these complications classified as major complications (Clavien grade ≥ 3). According to complications there were no significant differences between the groups. The overall complication rates were 17.6% in the G<20 and 14.4% in the G≥25 (p = 0.653). The major complication rates were 3.9% in the G<20 and 5.8% in the G≥25 (p=0.429). No differences in transfusion or urinary fistula rates were found.

**Conclusions:**

In this study, very thin patients were not at a higher risk of complications when submitted to PCNL than in those with a BMI of ≥25 kg/m^2^. Apparently, this technique can be used in these patients, just as it is used in any other type of patient, independently of their BMI.

## INTRODUCTION

Percutaneous nephrolithotomy (PCNL) is the gold standard treatment for large renal stones, according to the American, and the European guidelines (1–3). Nephrolithiasis has been associated to obesity in several epidemiologic studies (4, 5); therefore, several studies have evaluated the impact of high body mass index (BMI) on PCNL outcomes (6–8). However, there are no data evaluating the impact of a low BMI on PCNL complications.

According to some expert opinions, very thin patients are at a greater risk due to the lower perirenal adipose tissue, the higher kidney mobility, the retro-renal position of the colon, and even the lower functional capacity, which could predispose them to a higher complication rate. Currently, there are no studies in the literature investigating the outcomes of very thin patients undergoing PCNL in terms of complications and perioperative outcomes. The hypothesis is that these patients could have an increased risk of complications and worse outcomes from PCNL compared to non-thin patients.

This study aimed to evaluate if very thin patients are at higher risk of complications when submitted to PCNL in a single tertiary center.

## MATHERIALS AND METHODS

A matched case–control study was performed from June 2011 to October 2021 using a prospectively collected database of all patients who underwent PCNL. Informed consent was obtained from the patients, and the study protocol was approved by the local ethics committee (Institutional Review Board number: IRB: 8258117.8.0000.0091).

The patients were stratified according to their phenotypic characteristics, in two groups: very thin patients, arbitrarily defined as having BMI less than 20 kg/m2 (G<20) and non-thin patients, also arbitrarily defined as having a BMI equal or higher than 25 kg/m2 (Control group or G>25), in order to have two distinct groups regarding thinness. Patients were randomly matched based on Guy's Stone Score (GSS) according to case complexity at a ratio of 1:3.

The inclusion criteria were patients over 18 years old, with single or multiple renal stones >2 cm in size and symptomatic stones <2 cm in size, wherein first-line techniques (shockwave lithotripsy and ureteroscopy) failed. Patients excluded included pregnant women, patients with congenital or skeletal abnormalities, patients with refractory urinary tract infection, patients with coagulopathies, and those who refused to be included in the study. All patients underwent non-contrast computed tomography (CT) at least 6 months before the surgical procedure. Demographic data (age, gender, BMI, ASA score, and GSS) were analyzed. The GSS (9), routinely evaluated in all cases, was determined by a urologist during the preoperative consultation by CT scan analysis and was confirmed immediately before surgery. All urologists were trained in GSS, a nephrolithometry score known for its rapid application and reliable prediction of PCNL outcomes, compared to other nephrolithometry scores and nomograms (10–12). The intra- and post-operative data analyzed were operative time (defined as the time from cystoscopy until kidney drainage), fluoroscopy time, transfusion rates (intraoperatively and until discharge), tubeless approach (yes/no), complication rates, and length of hospital stay. The immediate success rate was defined as the absence of residual fragments >4 mm on CT scan performed in the first postoperative day (POD1). Complications were classified according to the Clavien-modified system, and complications with scores of ≥3 points were considered major complications (13).

### Surgical technique

All patients received general anesthesia during the procedure. The surgical technique was similar in all cases. Patients were placed in the prone or supine position, according to surgeons’ preference. A 6-Fr ureteral catheter was inserted through cystoscopy. After retrograde pyelography, the selected calyx was punctured under fluoroscopy guidance. Puncture was performed using an 18-gauge needle and a hydrophilic guidewire was inserted and passed through the ureter.

In cases in which multiple tracts were planned, all punctures and guidewire placements were performed prior to tract dilation. The tract was dilated using fascial dilators, and a 30 Fr Amplatz sheath was placed in all cases. A 26 Fr nephroscope (Karl Storz Germany^®^) and an ultrasonic device (Lithoclast Master, EMS^®^) were used for navigation and lithotripsy. An 18 Fr nephrostomy tube was placed at the end of the procedure in cases of bleeding, residual stones, renal pelvis perforation, or multiple accesses. In the absence of these findings, a double-J stent was placed for 2 weeks. The operation time was recorded from the beginning of cystoscopy to the end of nephrostomy tube placement or stent placement.

### Statistical analysis

Software R Core 3.5.1 was used for statistical analysis. Continuous variables were described by mean and standard deviations and were compared using Student's t-test. Categorical variables were described by simple and relative frequencies and were compared using the chi-square and Fisher's exact tests. Statistical significance was set at 0.05.

## RESULTS

A total of 204 patients were enrolled in this study: 51 patients (G<20) and 153 controls (G≥25). The median BMI was 27.23±2.81 Kg/m2, and the median age was 50.51±13.33 years. Complex stones (GSS 3 or 4) were 66.66% of the cases. The groups were similar according to demographic characteristics, being the BMI the only difference between the groups. The mean BMI was 18.43±1.03 Kg/m^2^ for G<20 and 30.29±4.60 Kg/m^2^ for G≥25, (p<0.001) (Table-1).

**Table 1:** Characteristics and demographic variables.

		G<20	G≥25	*P* value
		(n = 51)	(n = 153)	
**Gender; n (%)**				
	Male	23 (45.1)	61 (39.9)	0.516
	Female	28 (54.9)	92 (60.1)	
**Age (years)**				
	Mean (SD)	44.7 ± 14.4	49.6 ± 12.2	0.066
**BMI (kg/m^2^)**				
	Mean (SD)	18.4 ± 1.1	30.3 ± 4.6	<0.001
**ASA Score; n (%)**				
	I	23 (45.1)	42 (27.4)	0.473
	II	21 (41.2)	93 (60.8)	
	III	7 (13.7)	18 (11.8)	
**GSS; n (%)**				
	1	9 (17.7)	27 (17.7)	
	2	8 (15.7)	24 (15.7)	
	3	17 (33.3)	51 (33.3)	
	4	17 (33.3)	51 (33.3)	
Stone size (mm); mean (SD)		26.7 ± 15.1	27.2 ± 13.7	0.239

Data are presented as median (first quartile, third quartile) or number (proportion).

SD = standard deviation; BMI = body mass index; ASA = American Society of Anesthesiologists; GSS = Guy's stone score; HU: Hounsfield unit

Regarding operative variables, there were no statistically significant differences in the success rates, number of renal accesses, upper pole access, or operative time (Table-2).

**Table 2:** Operative variables.

		G<20	G≥25	*P* value
		(n = 51)	(n = 153)	
Operative time (min); mean (SD)		120.4 ± 46.6	121.21 ± 51.1	0.925
**Number of accesses; n (%)**				
	1	35 (68.6)	108 (70.6)	0.699
	2	13 (25.5)	32 (20.9)	
	3 or more	3 (5.9)	13 (8.5)	
**Upper calyx access**				
	n (%)	9 (17.6)	32 (20.9)	0.690
**Fluoroscopy time (min)**				
	Mean ± SD	14.92 (9.47)	14.42 (7.55)	0.735
**Tubeless**				
	n (%)	12 (23.5)	25 (16.3)	0.294
*Hospital stay (hour)*				
	Mean ± SD	67.53 (82.19)	63.90 (59.77)	0.772
Overall success rate; n (%)		19 (37.3)	52 (34)	0.735

Data are presented as median (first quartile, third quartile) or number (proportion).

SD = standard deviation

Complications were observed in 15.2% of the patients. Among the complications, 5.4% were major complications. There were no significant differences between the groups according to complications; overall complication rates were 17.6% and 14.4% in the G<20 and G≥25 groups, respectively (p=0.653), and major complications rates were 3.9% for G<20 and 5.8% for G≥25 (p=0.429). No differences in transfusion or urinary fistula rates were found (Table-3).

**Table 3:** Intra- and post-operative complications.

		G<20	G≥25	*P* value
		(n = 51)	(n = 153)	
Overall complication rate; n (%)		9 (17.6)	22 (14.4)	0.653
Major complication rate; n (%)		2 (3.9)	9 (5.8)	0.429
**Type of complication; n (%)**				
	Severe bleeding (transfusion)	2 (3.9)	8 (5.2)	0.728
	Urinary tract infection	1 (1.9)	5 (3.2)	
	Tract leakage (persistent fistula)	1 (1.9)	2 (1.3)	
	Pain	2 (3.9)	3 (1.9)	
	Stone migration to ureter	0 (0)	2 (1.3)	
	Acute kidney injury	1 (1.9)	1 (0.6)	
	Pleural injury	0 (0)	1 (0.6)	
	Bronchospasm	0 (0)	1 (0.6)	
	Hydrothorax	1 (1.9)	0 (0)	

Data are presented as number (proportion).

SD = standard deviation.

## DISCUSSION

Urolithiasis is one of the most common urological diseases and a frequent cause of morbidity and impaired quality of life worldwide (1–3). The management of urolithiasis has changed dramatically over the last three decades with the emergence of new technologies in endourology (2, 14, 15).

Obesity is a risk factor for the development of urinary stones, the role of a high BMI in treatment modalities for urolithiasis has been studied (7, 14, 17). The impact of obesity on PCNL does not seem to be important, since studies have shown that prone PCNL in normal-weight, obese, and super-obese individuals have similar outcomes (18, 19). In a publication of the CROES Percutaneous Nephrolithotomy Global Study a longer operation time, an inferior stone-free rate, and a higher re-intervention rate in obese patients were reported (20), however, this study did not standardize the PCNL technique. Ferreira et al. found no difference in outcomes and postoperative complications between obese and nonobese individuals who underwent a complete supine PCNL (8).

Conversely, there have been no comparative studies on how thinness may impact PCNL outcomes. Some endourologists have expressed concerns regarding PCNL in very thin patients, as they could carry a higher chance of complications due to difficult access linked to increased kidney mobility or a lack of perirenal fat. This could lead to poorer entrance orifice occlusion and, consequently, higher rates of bleeding or fistula formation. To the best of our knowledge, this is the first study to evaluate the impact of thinness on PCNL complications. We compared the data of 204 patients who underwent PCNL matched based on GSS at a ratio of 3:1. We arbitrarily selected BMI values of <20 based on the group's experience in visually classifying these patients as thin and associating this phenotype with a greater chance of complications. Conversely, patients with a BMI of ≥25 were visually classified as definitely non-thin, representing a different group from those with a BMI of <20, where potential surgical difficulties would not be encountered. All patients underwent a CT scan both before and after surgery, allowing surgeons to reliably evaluate their stone-free status and complications.

In the present study, the overall complication rate was low and not significantly different between thin and non-thin groups (17.6% and 14.4%, respectively, p=0.653), and major complications were predominant in the control group (40.9%, p=0.429). There was, also, no significant difference in the immediate success rate between the two groups (37.3% vs. 34.0%, p=0.735). A stone size of ≤4 mm was used as the threshold to determine immediate success. It has been found to be a cost-effective threshold for the management of patients with residual fragments after PCNL (21). A POD1 CT scan ensured a high level of imaging accuracy. Vicentini et al., in a large descriptive study validating GSS involving more than 1,000 PCNL procedures, reported that the stone-free rate was inversely proportional to stone complexity, with GSS grades 1, 2, 3, and 4 having stone-free rates of 85%, 60%, 45%, and 25%, respectively (22). The high number of complex stones in our series (approximately 66% GSS of 3 and 4) is consistent with the observed stone-free rates in our patients (23).

Certain aspects may differ between thin and obese patients who have undergone PCNL. Based on our experience, we advocate for specialized care for this group of patients. Kidney movement during puncture seems to be more pronounced when patients are in a supine position, and it is not uncommon to manually stabilize the kidney while dilating it by applying pressure to the medial side with the hand not holding the needle. A smaller sheath caliber appears to be more suitable for these patients, as they typically have a lower total blood volume. Using a sheath caliber greater than 24 Fr is associated with a more significant decrease in hemoglobin levels (24). In these patients, it is important to have the sheath adequately inserted inside the calyx to avoid perirenal liquid leakage due to lack of fat for blockage (25). Nephrostomy tubes do not seem to avoid fistula, and it is not indicated as usual for any patient.

Our study has some limitations. First, this was a retrospective study, despite the database being collected prospectively, and a matched-paired comparison was performed to decrease confounders. Second, the number of enrolled patients was relatively small to draw strong conclusions. At the time of the study, miniaturized PCNL, endoscopic combined intrarenal surgery or ultrasound-guided puncture were not routinely performed at our institution, and some endpoints could be different today, reducing bleeding complications and the fluoroscopy time (24, 26). In this study a 30 Fr accesses were performed for the use of a 26 Fr nephroscope.

Until more studies with a higher number of enrolled patients are available, our study does not support the impression that thinness has a negative impact on the PCNL outcomes.

## CONCLUSIONS

Thinness (BMI less than 20 kg/m^2^) was not associated with higher complication rates in patients who underwent PCNL compared to those with a BMI of 25 kg/m^2^ or more. This technique appears to be safely applicable in very thin patients.

## ABBREVIATIONS

PCNL: = Percutaneous nephrolithotomy
BMI: = Body Mass Index
G<20: = Very thin patients, body mass index <20 kg/m^2^
G≥25: = Non-thin patients, body mass index ≥25 kg/m2
GSS: = Guy´s Stone Score
ASA: = Anesthesiologists physical status classification
CT: = Computed tomography
POD1: = First postoperative day

## Availability of data and material

All data are filed in a database (REDCap^®^ software).
